# Chiral Motifs in Highly Interpenetrated Metal–Organic Frameworks Formed from Achiral Tetrahedral Ligands**

**DOI:** 10.1002/chem.202201108

**Published:** 2022-08-03

**Authors:** Qiang Wen, Maria Chiara di Gregorio, Linda J. W. Shimon, Iddo Pinkas, Naveen Malik, Anna Kossoy, Eugeny V. Alexandrov, Davide M. Proserpio, Michal Lahav, Milko E. van der Boom

**Affiliations:** ^1^ Department of Molecular Chemistry and Materials Science Weizmann Institute of Science Rehovot 7610001 Israel; ^2^ Department of Chemical Research Support Weizmann Institute of Science Rehovot 7610001 Israel; ^3^ Samara Center for Theoretical Materials Science (SCTMS) Samara State Technical University Samara 443100 Russia; ^4^ Samara Branch of P. N. Lebedev Physical Institute of the Russian Academy of Sciences Samara 443011 Russia; ^5^ Dipartimento di Chimica Università degli Studi di Milano 20133 Milano Italy

**Keywords:** channels, diamondoid networks, interpenetration, metal–organic frameworks, symmetry breaking

## Abstract

Formation of highly interpenetrated frameworks is demonstrated. An interesting observation is the presence of very large adamantane‐shaped cages in a single network, making these crystals new entries in the collection of diamondoid‐type metal–organic frameworks (MOFs). The frameworks were constructed by assembling tetrahedral pyridine ligands and copper dichloride. Currently, the networks’ degree of interpenetration is among the highest reported and increases when the size of the ligand is increased. Highly interpenetrated frameworks typically have low surface contact areas. In contrast, in our systems, the voids take up to 63 % of the unit cell volume. The MOFs have chiral features but are formed from achiral components. The chirality is manifested by the coordination chemistry around the metal center, the structure of the helicoidal channels, and the motifs of the individual networks. Channels of both handednesses are present within the unit cells. This phenomenon shapes the walls of the channels, which are composed of 10, 16, or 32 chains correlated with the degree of interpenetration 10‐, 16‐, and 32‐fold, respectively. By changing the distance between the center of the ligand and the coordination moieties, we succeeded in tuning the diameter of the channels. Relatively large channels were formed, having diameters up to 31.0 Å×14.8 Å.

## Graphical abstract

**Tetrahydral achiral ligands** and copper ions assemble into a record number of interpenetrating diamondoid networks. The networks intertwine akin to strands of a molecular rope to shape the chiral channels. The design of the ligand “arms” allows modulating their degree of interpenetration and the channel properties. The concurrent presence of such numerous interpenetrated networks and porosity is unprecedented for metal–organic frameworks.

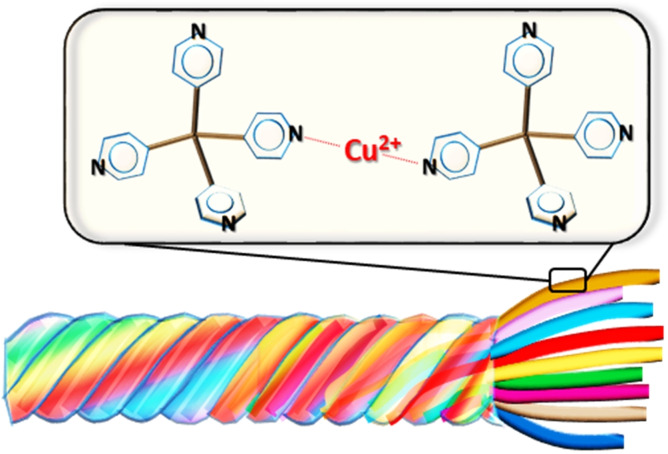

## Introduction

Chirality is a key element of life and a desirable property in many functional materials. Better understanding how it evolves and recognizing when it is present are important, both for inferring general rules of fundamental interest and for enlarging the library of potential useful materials.[^1^, ^2^, ^3^, ^4^, ^5^] Among the synthetic systems, metal–organic frameworks (MOFs) are crystalline coordination polymers formed by metallic nodes and organic ligands as connectors among the nodes.^[6]^ The metal‐ligand interaction leads to molecular packing, which may be porous and where both continuous channels and 3D cages can be present. In this class of material, the chirality is mainly analyzed at the interior surface of pores and channels due to the possibility to exploit these areas for enantiomeric separation and chiral catalysis.[^7^, ^8^, ^9^] When enantiopure chiral ligands are used, MOFs are packed into Sohncke or chiral space groups.[^10^, ^11^, ^12^] Such crystals often exhibit homochiral channels and are homochiral bulk solids. Interestingly, chiral MOFs can also be formed from achiral components. For example, Morris et al. demonstrated how solvents can induce homochirality in MOFs.^[13]^ Others have observed that racemic mixtures, chiral additives, and even impurities can yield chiral crystals.[^14^, ^15^] Such studies serve as relevant models for better understanding symmetry breaking in nature and in studies aimed at revealing the origin of life.[^16^, ^17^] Despite the numerous studies in this area, designing chiral MOFs from achiral compounds without using additives remains a challenge. The possibility that one could also use achiral ligands to design chiral MOFs without the use of additives increases the pool of available ligands. Likewise, it is also difficult to predict a combination of structural properties such as porosity, network interpenetration, stability, morphology, and surface properties.[^18^, ^19^, ^20^] For example, diamondoid‐type structures have been demonstrated by coordination chemistry; they can display a rich isomerism of interpenetrating networks.[^21^, ^22^] The degree of interpenetration is related to the degree of porosity, which in turn, controls functionalities including guest absorption, catalysis, and sensing.[^23^, ^24^] Yang, Batten, Ma, and co‐workers reported an extraordinary interpenetrated MOF consisting of a 54‐fold interpenetrated coordination framework.^[25]^ The characteristics of known structures with highly interpenetrated underlying **dia** nets are summarized in Figure S1 and Table S1.

We previously reported that tetrahedral pyridine‐based ligands generate metallo‐organic single crystals having a uniform and/or unusual multidomain morphology.[^26^, ^27^] Their growth, starting from these achiral ligands and metal salts, involves solvothermal processes and results in the formation of chiral molecular packing. The crystals are isostructural, regardless of the nickel or copper salts used; they exhibit two different types of homochiral channels. The framework is based on the coordination of four pyridine groups in a propeller‐like arrangement around the metal cations. The relationship between the achiral tetrahedral pyridine ligands, chiral molecular networks, and channel properties is complex.

In this study, the coordination chemistry of three different but structurally related ligands with copper dichloride is reported. The structures we obtained confirm the tendency of this kind of ligand to generate chiral channels.[^26^, ^27^, ^28^] These channels are formed by an unprecedented number of individual networks; the overall structures are among the highest reported interpenetrating frameworks. The combination of highly interpenetrated networks and continuous channels is not common; it results in high contact surface areas. Another fascinating structural aspect is the presence of huge adamantane cages.

## Results and Discussion

**Figure 1:**
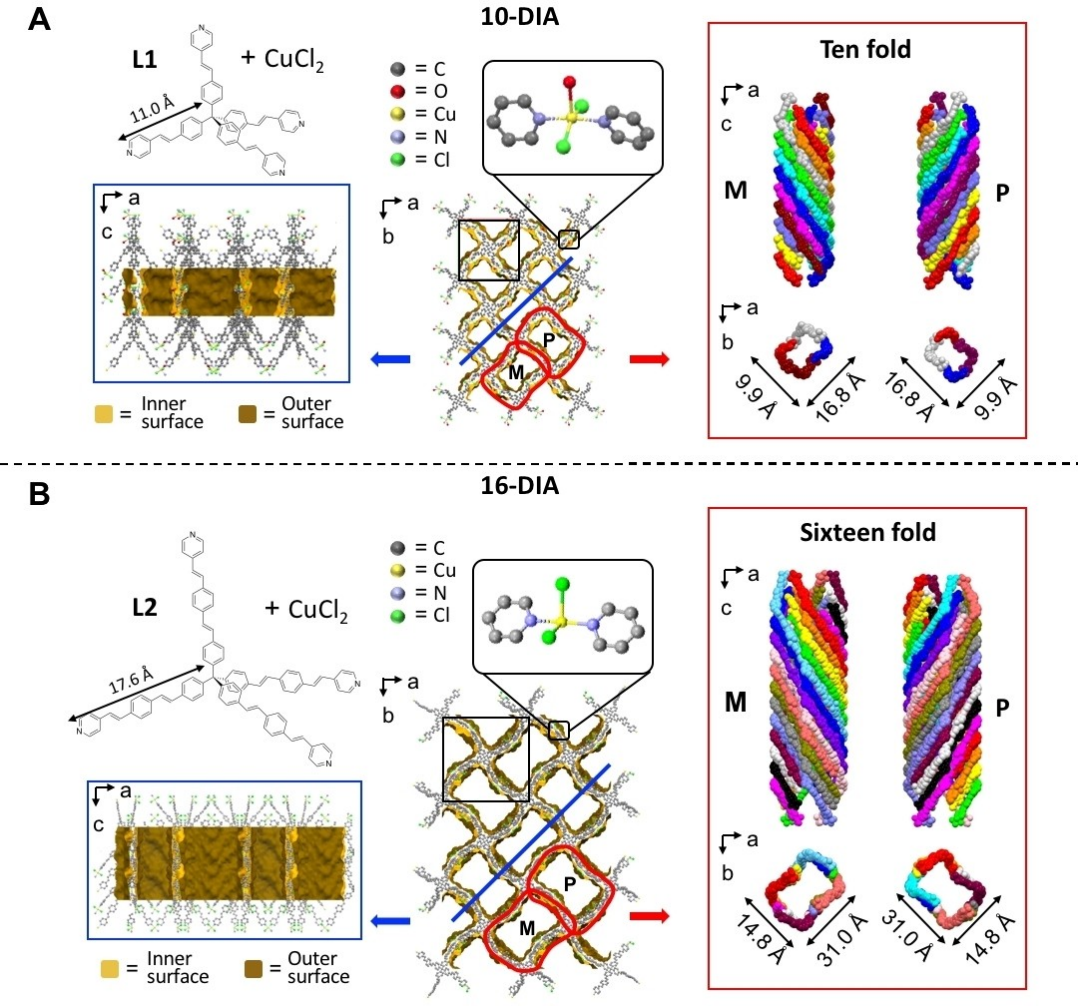
Single crystal X‐ray structures of **10‐DIA** (A) and **16‐DIA** (B). Building blocks (**L1**, **L2**) are shown in the top left corners. Connolly surface representations of the crystal structures appear along the b (left, blue frames) and c (center) axes. The channels and their helicoidal packing (right) are denoted by red frames. The zoom‐in views show the coordination centers. Two of the four half‐occupancy Cl atoms for the disordered sites for **16‐DIA** are shown.

**Figure 2:**
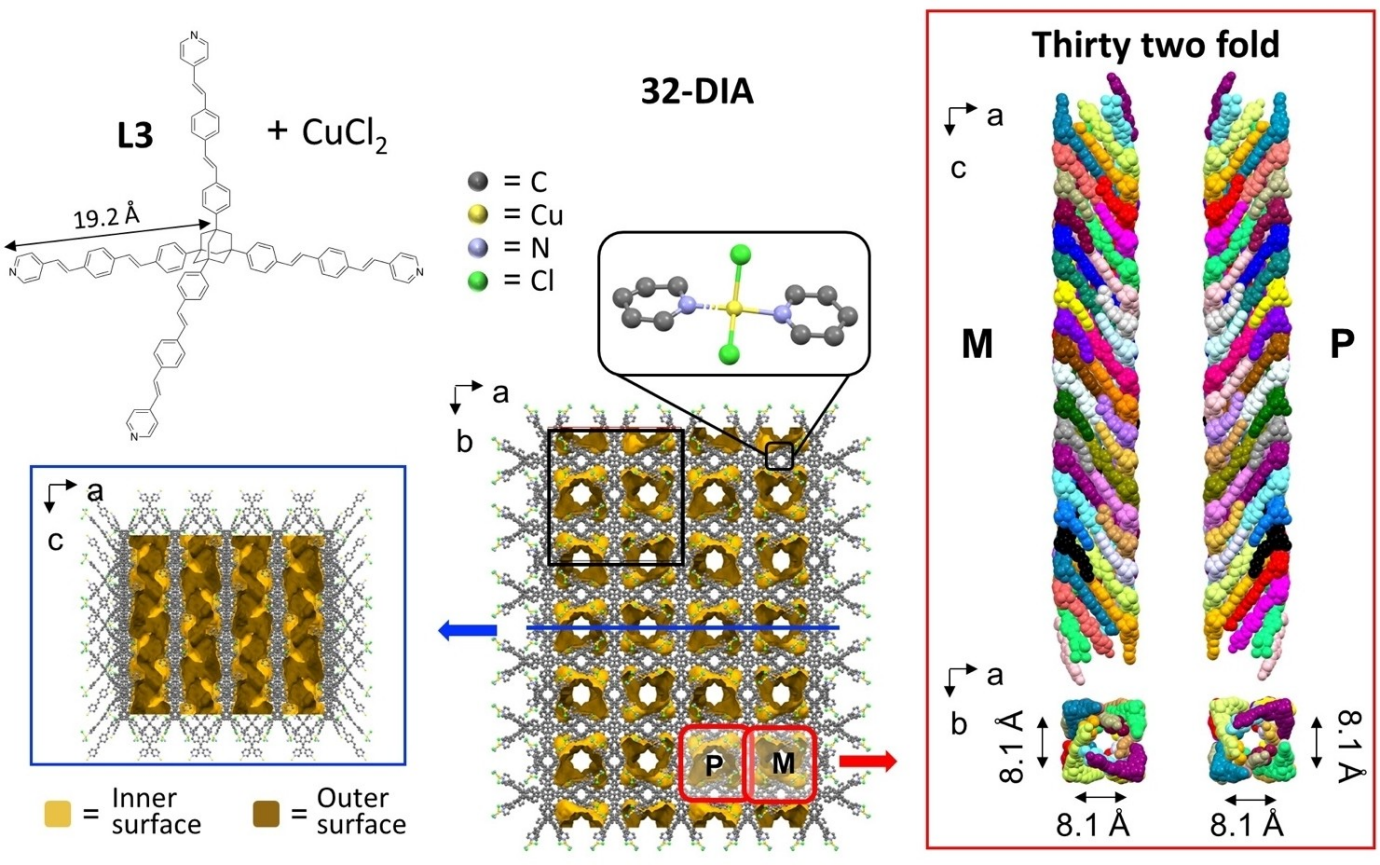
Single crystal X‐ray structure of **32‐DIA**. The building block (**L3**) appears in the top left corner. Connolly surface representations of the crystal structure appear along the b (left, blue frame) and c (center) axes. The channels and their helicoidal packing (right) are denoted by red frames. The zoom‐in view shows the coordination center (top center).

The ligands (**L1‐L3**) we used are achiral, and owing to their tetrahedral symmetry (*T*_d_), they are structurally suitable for the general strategy for forming diamondoid‐type structures.^[29]^ In 1989 Robson and co‐workers used this reticular approach to construct diamondoid structures by coordinating cyano groups with metal centers.^[30]^ Structurally, our ligands comprise four pyridine moieties and a carbon (**L1** and **L2**) or an adamantane (**L3**) core (Figure 1 and 2, top left corner; Scheme S1). The distance between the pyridine moieties of consecutive “arms”, and the distance between the pyridine moieties and the core can be varied by using vinyl‐phenyl groups. Yaghi and Schröder showed that elongation of the “arms”, both in branched and linear ligands, is a strategy used to increase the dimensions of channels in MOFs.[^31^, ^32^] The coordinative chemistry of pyridine and divalent cations such as Cu^2+^ has been well established, and it is used to construct sophisticated assemblies, demonstrated by Sauvage,^[33]^ Fujita,^[34]^ and others.[^35^, ^36^, ^37^, ^38^] The three MOFs reported here were grown using copper dichloride, **L1‐L3**, under solvothermal reaction conditions (DMF, T=105 °C). The vessels were gradually cooled down after 16–48 h of heating. In order to solubilize the ligands in DMF, we used small amounts of HCl, which, by protonating the pyridine groups, enhances their polarity. This method may seem counterintuitive, as the coordination sites are actually inactivated; however, the formation of coordination‐based crystals is thermodynamically favored. The materials were analyzed by both bulk and single crystal measurements: optical microscopy, microRaman and UV/Vis spectroscopy, elemental and thermogravimetric analysis (TGA), Powder X‐ray Diffraction (PXRD), and single‐crystal X‐ray Diffraction (SCXRD) measurements. Optical microscopy images show that **10‐DIA** consists of green rod‐like structures (Figure S2). Green rods and multibranched aggregates were observed for **16‐DIA** (Figure S3). The sample obtained from the reaction between **L3** and CuCl_2_ contains **32‐DIA** as globular reddish structures and needles of **L3**, as judged by optical (Figure S4) and microRaman spectroscopy (Figures S5, S6). Attempts to remove **L3** by washing in organic solvents were unsuccessful because of its low solubility.

MicroRaman spectra were collected on single crystals of **10‐DIA**, **16‐DIA**, and **32‐DIA**. Major variations with respect to the ligand spectra (**L1**, **L2**, and **L3**) were observed both in the position and ratio of the peaks in the *ν*=1590–1633 cm^−1^ range, indicative of pyridine coordination to the metal centers.^[39]^ UV/Vis absorption bands of dried samples span from *λ*=370 up to the near IR region. The spectroscopic features indicate the presence of both ligand‐to‐metal and metal‐to‐ligand charge transfer (Figure S7).[^40^, ^41^] Use of thermogravimetric analysis (TGA) under nitrogen reveals comparable properties for **10‐DIA** and **16‐DIA**, in agreement with the similarity in the porosity and the single network structure (see below) (Figure S8). Less pronounced steps for loss of solvents and moisture are observed for samples containing both **32‐DIA** and **L3**, likely due a lower porosity and the presence of **L3**. The discrepancy between the elemental analysis of the bulk and the formula derived from the SCXRD data of both **10‐DIA** and **16‐DIA** might indicate solvent inclusion in the crystals, as demonstrated by the TGA analysis, in addition to the presence of unreacted starting materials (Table S2). TGA‐GCMS measurements show the presence of DMF in both **10‐DIA** and **16‐DIA**. The sample of **10‐DIA** also includes ethanol (used for washing) (Table S2). Solvent inclusion is common for MOFs.^[42]^ PXRD measurements carried out in the presence of DMF for **10‐DIA**, **16‐DIA**, and **32‐DIA** demonstrate that the bulk is crystalline (Figure S9). However, the compounds are unstable, as shown by repetitive scanning. Most likely the framework collapses due to loss of solvent molecules.

**Figure 3:**
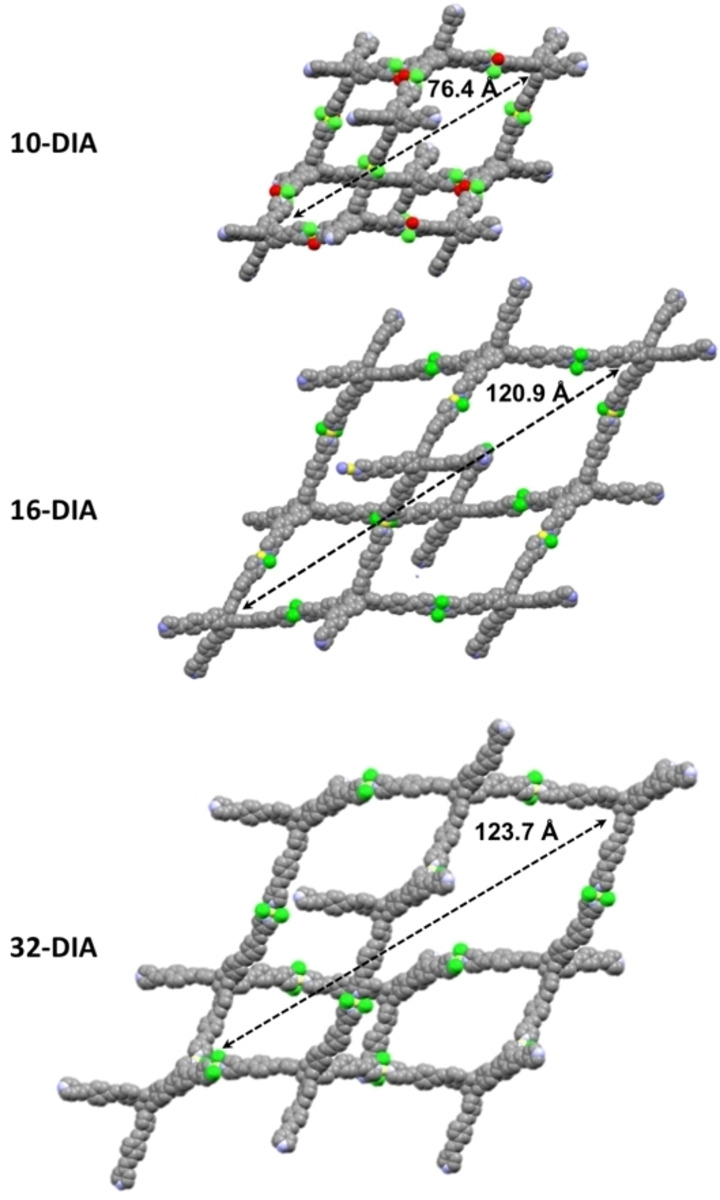
Space‐filling representation of the adamantane‐type cage motifs characterizing the constitutive networks of **10‐DIA** (top), **16‐DIA** (center), and **32‐DIA** (bottom). The color code is yellow for copper, red for oxygen, green for chlorine, violet for nitrogen, and gray for carbon.

Single‐crystal X‐ray analysis revealed that **10‐DIA** and **16‐DIA** crystallize in non‐centrosymmetric space groups *P*‐4 and *P*‐4*n*2, whereas **32‐DIA** crystallizes in a centrosymmetric space group, *I*4_1_ */acd* (Table S3). For these three MOFs, the framework structures consist of highly interpenetrated diamondoid‐based networks (Figure S10 and S11). These networks are formed by coordinating two pyridine moieties belonging to two different ligands with Cu(II)^[43]^ (Figure 1 and 2, top center). Despite of the different anions and co‐ligands (Cl^−^, H_2_O), the structures of the constitutive networks are characterized by adamantane‐type cage motifs (Figure 3).

Two sets of channels are present by a helical arrangement of the conjugated vinyl‐pyridine moieties. The walls of the channels have an opposite chirality (*P* and *M*) and run along the *c*‐axis (Figures 1 and 2, right). The crystals grown from **L1** and **L2** have a very similar structural appearance (e.g., the shape and disposition of the channels) in comparison with **32‐DIA**. For **10‐DIA** and **16‐DIA**, the channels run along the 4‐fold roto‐inversion axis and for **32‐DIA** the channels are aligned along the 4_1_ screw axis. The channels of **10‐DIA** and **16‐DIA** are rectangular, having apertures of 16.8×9.5 Å and 31.0×14.8 Å, respectively, whereas the channels of **32‐DIA** are square when viewed along the c‐axis (8.1×8.1 Å). Analysis by ToposPro^[44]^ revealed that the number of networks, i.e., the degree of interpenetration is 10, 16, and 32 for **10‐DIA**, **16‐DIA**, and **32‐DIA**, respectively (Figure S12, Table S1).

The interpenetration modes were described by Baburin et al. in 2005 using a geometrical approach according to three classes.^[45]^ In 2012 Alexandrov et al. used topological types to compute the Hopf ring nets, an approach that is independent of the geometrical embedding: different classes may be observed for the same topological type.^[46]^ Diamondoid nets are among the most frequently observed underlying nets[^47^, ^48^] and their modes of interpenetration are enumerated for the maximum symmetry embedding, that is, with exact tetrahedral nodes, straight edges and transitivity [11] (one kind of node and one kind of edge).[^49^, ^50^] The comparison with the observed structures indeed shows that the smallest transitivity (the number [*pq*] of inequivalent nodes *p* and edges *q*) is the one actually observed (Figure S12). **10‐DIA** is 10‐fold interpenetrated with class Ia, i.e., the ten nets are related by a single translation [001] that makes the ten nets eclipse if viewed along the *c*‐axis. **16‐DIA** is 16‐fold of the class IIIa, where the translation vector [001] relates to 8 interpenetrating networks, and the 8 other networks are related to former ones by two diagonal glide planes *n* oriented as (100) and (010). What is peculiar here is that this geometry does not affect the topological type of entanglement, which is the same as the one observed from **10‐DIA**, where all the nets are related by a single translational vector. We can conclude that the two structures both present the most common interpenetration mode that aligns all nets equally spaced along the S_4_‐axis of the adamantane cage (the so‐called “normal mode”, first described in 1998 (Figure S13).[^51^, ^52^, ^53^] **32‐DIA** is 32‐fold interpenetrated with the rarely seen class IIIc (Figures S1, S12, Table S1), related by four translations and three non‐translational symmetry elements *i*, C_2_, and glide *d*. Interestingly, all the networks participate in the structure of the channel walls, akin to the strands of a rope. Complete details on the classes are reported in the Supporting Information (Figure S1, Table S1).

**Figure 4:**
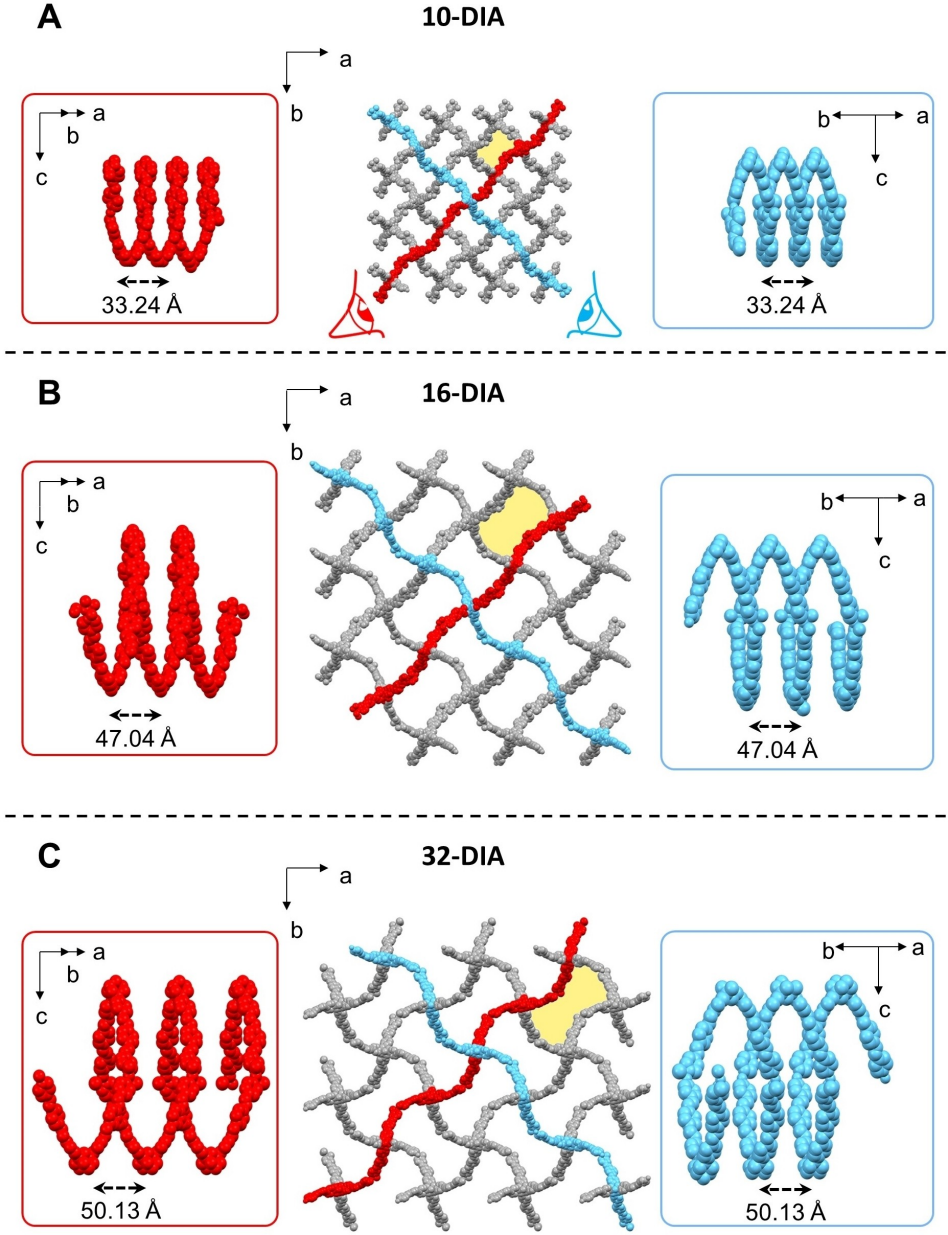
Structure of one network constituting **10‐DIA** (A), **16‐DIA** (B) and **32‐DIA** (C). These networks (red and cyan frames) are characterized by having helicoids of opposite handedness.

Further analysis reveals that the three MOFs are fabricated by structurally similar individual networks having different dimensions. Viewed from the S_4_‐axis, the adamantane‐type cages are distorted, which increases when moving from **10‐DIA** to **32‐DIA**. Interestingly, these networks are made by perpendicularly positioned helicoids having opposite handedness (Figures 4, S14 and S15). The overall crystal structures of the MOFs appear different because the interpenetration masks the structures of the individual networks, especially for **32‐DIA**.

The largest dimension of the adamantane‐type cages also increases from 76.4 Å (**10‐DIA**) to 120.9 Å (**16‐DIA**); however, the cage dimensions for **16‐DIA** and **32‐DIA** are nearly identical (120.9 Å vs. 123.7 Å). The number of interpenetrating networks for **32‐DIA** is remarkably high, indicating that varying the ligand core consistently affects the MOF packing. From **10‐DIA** to **16‐DIA**, the calculated porosity increases from 50.9 % to 64.1 % of the unit cell volume. The porosity decreases in **32‐DIA** (28.6 %) due to the different mode of interpenetration. Note that the three modes involve transitivity [11] (the topologically simplest structure is the one obtained); 15 of the 17 known examples of diamondoid nets have 10‐fold or more interpenetration, as presented in the Table S1. The 10‐fold structures with CSD refcodes, FUXCOS, PUQDOW, XISXAY, and MOXPUL, and the 12‐fold structure, OMUNER09, have similar 1‐periodic helical channels of rectangular cross‐section, as observed in **10‐DIA** and **16‐DIA** because of a similar “normal mode” of interpenetration. From the analysis of the three structures, we can see that the record 32‐fold interpenetration of **dia** nets in **32‐DIA** arises from the longer ligand bridged with metal cations (**L1**: 11.0 Å, **L2**: 17.6 Å, **L3**: 19.2 Å). Bridging metal cations elongating the edges also gives rise to other structures with the highest degrees of interpenetration 20 (KUVMEV) and 25 (ZEFHEZ). Some loose correlation could be seen relating to the edge length and the angles at the mid edge, as shown in the plots for the 20 structures (Figure S16). The size of the accessible channels is also not always proportional to the elongation of linkers. Some of the straight edges of the underlying net of **32‐DIA** intersect due to an abnormal mutual orientation of nets; therefore, the walls of the channels are constructed using bent linkers (angle 147°) (Figure S17). In this way, the observed mode of interpenetration (abnormal) reduces the pore dimensions more than the normal mode would and increases the density of the structure due to the larger number of nets filling the same space. The abnormal mode of interpenetration also prevents the presence of accessible channels in six other highly interpenetrating structures (FOKZEM, VOTQAY, WODYIZ, NUCJEB, ZEFHEZ, and KUVMEV). How do the crystal structures for the MOFs comprising **L1**, **L2**, and **L3** compare? The obvious differences lie in the core and in the “arm” dimensions. Looking at the ligands in the crystal packing, one can observe the following characteristics: All the ligands have similar distortions. Starting from symmetric pyramids (equilateral triangles as facets), where the ligands are inscribed with the pyridine units at the vertexes, deviations occur in asymmetric pyramids with isosceles triangles as facets (Figure S17). The ratio between the sides of the triangles is 1.3‐1.5. The triangle angles are about 43° and 68°. The volumes inscribing **L1**, **L2**, and **L3** are 560.4, 2152.5, and 2895.1 Å^3^, respectively. The “arms” of **L3** are more curved. We noted that for both **L2** and **L3** the pyridine groups are rotated either oppositely or coherently with respect to the phenyl rings. This results in two sets of two structurally different “arms” within one molecule. However, this effect is not observed in **L1**. Addressing the posed question is not trivial. The adamantane cage size, interpenetration level, and ligand curvature (bending) appear to be correlated. The increased size of the adamantane cages allows for a higher interpenetration of the networks. This structural effect is consistent with a higher bending of the ligand arms.

## Conclusion

The chirality of the distorted diamondoid metal–organic frameworks is expressed at different levels in these crystals, as the constitutive networks themselves are helicoids. Crystals having opposite chirality within the same unit cell were formed. Previously we have produced crystals with two types of homochiral channels.[^26^, ^27^, ^28^] Changing the ligand core (carbon vs. adamantane) drastically changes the overall appearance of the framework, including the shape of the channels. However, changing only the length of the “arm”, while maintaining the C core, affects the diameter of the channels while preserving the shape. It is interesting to note that the individual networks for the three MOFs are similar in shape; however, the interpenetration levels and modes are different. A high level of interpenetration usually goes hand in hand with low solvent accessible surface and contact surface areas; however, the opposite was observed in our MOFs. In spite of the increasing level of interpenetration, the crystals still contain continuous channels, even in **32‐DIA**, with 32 interpenetrating networks and 28 % porosity. The presence of the channels explains the relatively high porosity of these crystals. For example, 25‐fold^[22]^ and 54‐fold^[25]^ interpenetrated MOFs have lower calculated solvent accessible surface and contact surface areas. A 32‐fold interpenetrated hydrogen bond^[54]^ assembly has a comparable porosity (Table S4).

The achiral tetrahedral ligands used in this and other related studies appear to have a general tendency to form assemblies with chiral channels, regardless of the crystallization conditions (acidic vs. neutral conditions, Cu^2+^ vs. Ni^2+^, counter ions), and ligand structure (core, bond order, and “arm” length).[^26^, ^28^] The channels’ diameter, shape, and the nature of their chirality (homochiral vs. heterochiral) appear to be a function of the ligand structure. Such structures with different channel topologies provide opportunities for selective inclusion of guest molecules.^[55]^ Moreover, the perpendicularly positioned chiral motifs have opposite handedness (Figure 4), indicating that such crystals can have interesting directional chiroptical properties.[^56^, ^57^]

## Experimental Section

**Chemicals and reagents**: Chloroform (CHCl_3_, ≥99.8 %), dimethylformamide (DMF, ≥99.8 %), and copper(II) chloride (CuCl_2_, 97.0 %) were purchased from Sigma Aldrich. Reagents were used without further purification. **L1** was synthesized according to a literature procedure.^[58]^

**Optical microscopy**: Optical microscopy images were collected with a Nikon Eclipse LV100ND microscope. Image processing for extended focus imaging was performed by DeltaPix 2001–2022 v6.5.3.

**MicroRaman spectroscopy**: Raman measurements were performed on a LabRAM HR Evolution instrument (Horiba, France), equipped with an 800 mm spectrograph and a CCD detector (1024 pixels×256 pixels open electrode front illuminated CCD camera, cooled to −60 °C). The system is based on an open confocal microscope (BX‐FM Olympus, Japan). A 632.8 nm HeNe laser with 600 grooves/mm grating was used for the measurements together with a x100 or x150 objective NA=0.9 WD=1 mm, MPLanFL N and MPLanFL N BD respectively (Olympus Japan). The pixel spacing under these conditions is about 2 cm^−1^.

**UV/Vis absorption**: UV/Vis absorption measurements were performed with a HAMAMATSU Absolute PL Quantum Yield Spectrometer C11347. The instrument is equipped with a 150 W Xenon light source and an integrating sphere made of 3.3 inch Spectralon. Crystals were separated by centrifugation, washed with ethanol and dried under vacuum overnight. The samples were weighed before the measurements. The spectra were collected using 3.2 mmol of the materials, in the range of the 370–800 nm, with steps of 10 nm.

**Thermogravimetric analysis (TGA)**: TGA was performed by a SDT Q600 V8.3 Build 101 Instrument. Crystals were separated by centrifugation, washed with methanol and dried under vacuum overnight. The measurements were performed under a stream of nitrogen. The samples were placed in an alumina crucible.

**Powder X‐ray diffraction (PXRD)**: The measurements were performed at ambient conditions on TTRAX‐III (Rigaku, Japan) diffractometer equipped with a rotating Cu anode operating at 50 kV and 200 mA in Bragg‐Brentano mode. A suspension of crystals was drop‐cast onto a Si zero‐background stage and measured 3 times with a scan speed of 1 degree/min. The interval between measurements was ∼38 min.

**Wide angle X‐ray scattering (WAXS)**: Quartz capillaries were used to measure samples of **10‐DIA**. The crystals in the original solution were briefly centrifuged and were crushed. The measurements were performed on SmartLab (Rigaku, Japan) diffractometer equipped with a rotating Cu anode operating at 45 kV and 200 mA and with a HyPix‐3000 two‐dimensional detector. A quasi‐parallel X‐ray beam was formed by a multilayer mirror (CBO attachment, Rigaku), and then collimated with 0.3 mm pinhole and 0.1 mm collimator. In addition, 1 mm pinhole was positioned before the sample. The distance between the sample and the detector was between 25–40 mm.

**Elemental analyses**: Elemental analyses (C, H, N, Cl, Cu) were performed at Kolbe Laboratorium, Mulheim, Germany. The oxygen is calculated as the leak to 100 %. The errors are ±0.01 % for C, H, N; ±0.015 % for Cu and Cl.

**Single‐crystal X‐ray diffraction (SCXRD) analysis**: A single crystal was coated in Paratone oil (Hampton Research) and mounted on MiTeGen loops. It was flash frozen in the liquid nitrogen stream of the Oxford Cryostream. Diffraction data were measured at a low temperature of 100(2) K using Cu K*α λ*=1.54184 Å on a Rigaku XtaLab^Pro^ diffractometer equipped with a Dectris Pilatus 3R 200K−A detector. The Rigaku data were processed and reduced with ′CrysAlisPro 1.171.39.46 (Rigaku OD, 2018)′. The structure was solved with SHELXT‐2016/4 and refined with SHELXT‐2016/4.^[59]^ All non‐hydrogen atoms were refined anisotropically and hydrogens were placed in calculated positions and refined in riding mode. The SQUEEZE protocol of Platon^[60]^ was run on all structures. The crystal data and the structural refinements are summarized in Table S3.

## Preparation of ligands L2 and L3

*Synthesis of (E)‐4‐(4‐vinylstyryl)pyridine* (**3**): Compound **2** ^[61]^ (3.5 g, 14 mmol) was dissolved in 60 mL of dry THF. Subsequently *t*‐BuOK (2.68 g, 24.0 mmol) and compound **1** (1.07 g, 10.0 mmol) were gradually added while keeping the temperature of the reaction vessel at 0 °C. The mixture was stirred at 0 °C for 30 min. Then, the reaction mixture was allowed to reach room temperature and was stirred for an additional 12 h. The solvent was removed by evaporation and the obtained solid was re‐dissolved in 100 mL of H_2_O. An extraction was performed with CH_2_Cl_2_ (3×100 mL). Subsequently the organic extracts were combined, dried with MgSO_4_ and evaporated to afford a white solid. After purification with flash column chromatography on silica gel (hexane/ethyl acetate, v/v, 3/10‐1/1), a white solid was obtained (1.5 g; 72 % yield based on **1**). For alternative procedures see Ref. [62, 63]. ^1^H NMR (500 MHz, CDCl_3_) δ 8.58 (d, *J*=3.2 Hz, 2H), 7.51 (d, *J*=8.1 Hz, 2H), 7.43 (d, *J*=8.1 Hz, 2H), 7.36 (d, *J*=5.6 Hz, 2H), 7.29 (d, *J*=16.3 Hz, 1H), 7.01 (d, *J*=16.3 Hz, 1H), 6.73 (dd, *J*=17.6, 10.9 Hz, 1H), 5.80 (d, *J*=17.6 Hz, 1H), 5.29 (d, *J*=10.9 Hz, 1H). ^13^C NMR (126 MHz, CDCl_3_) δ 150.18, 144.55, 137.99, 136.22, 135.60, 132.69, 127.20, 126.65, 125.80, 120.80, 114.47. ESI‐MS: *m*/*z* [M+H]^+^ calcd. for C_15_H_14_N: 208.28, found: 208.18, correct isotope distribution.

*Synthesis of **L2***: Compound **4** ^[64]^ (236 mg, 0.25 mmol, 1.0 equiv.), NaOAc (123 mg, 1.5 mmol, 6 equiv.), Hermann's catalyst (20 mg, 0.021 mmol, 0.08 equiv) and 4[4‐ethenyl‐(2‐(*E*)‐phenylethenyl)]pyridine (415 mg, 2.0 mmol, 8 equiv) were mixed in a 50 mL pressure tube. Subsequently dry *N*‐methyl‐2‐pyrrolidone (6 mL) was added in a N_2_ filled glovebox. The pressure tube was closed and heated in an oil bath at 130 °C for 5 days. After cooling down the tube, 30 mL of H_2_O was added and the resulting solid was collected via filtration. The solid was washed with water and dried under vacuum. Acetone (300 mL) was used to disperse the solid which was then filtered. The product (**L2**) was obtained by collecting the remaining solid which was further purified by flash column chromatography. The silica was first neutralized with triethylamine and then eluted with CHCl_3_ mixed with methanol (1.0‐4.0 %) resulting in the isolation of **L2** (160 mg, yield, 51 %). ^1^H NMR (500 MHz, CDCl_3_) δ. 8.45 (d, *J*=5.8 Hz, 8H), 7.49 (s, 16H), 7.41 (d, *J*=8.3 Hz, 8H), 7.36 (d, *J*=5.8 Hz, 8H), 7.28 (d, *J*=16.3 Hz, 4H), 7.24 (d, *J*=10.4 Hz, 8H), 7.11 (d, *J*=16.3 Hz, 4H), 7.05 (d, *J*=16.3 Hz, 4H), 6.98 (d, *J*=16.3 Hz, 4H). ^13^C NMR (126 MHz, CDCl_3_) δ 149.40, 146.09, 145.23, 137.88, 135.20, 134.86, 133.14, 131.19, 128.70, 127.98, 127.38, 126.83, 125.83, 125.31, 120.98. UV/Vis (CHCl_3_): *λ*_max_ (ϵ, cm^−1^ M^−1^)=367 nm (1.31×10^5^). ESI‐MS: *m*/*z* [M+H]^+^ calcd. for C_85_H_65_N_4_: 1142.45, found: 1142.77, correct isotope distribution.

*Synthesis of **L3***: Compound **5** ^[65]^ (236 mg, 0.25 mmol), NaOAc (123 mg, 1.5 mmol, 6 equiv.), Hermann's catalyst (20 mg, 0.021 mmol, 0.08 equiv.), and 4[4‐ethenyl‐(2‐(*E*)‐phenylethenyl)]‐pyridine (415 mg, 2.0 mmol, 8 equiv.) were added in a 50 mL glass pressure tube. Dry *N*‐methyl‐2‐pyrrolidone (6 mL) was added in a N_2_‐filled glovebox. The sealed tube was heated for 130 °C for 5 days. After cooling down, 30 mL of H_2_O were added and the resulting precipitate was collected via filtration. The collected solid was washed with water and dried under vacuum. Acetone (300 mL) was used to disperse the solid which was then filtered. The product (**L3**) was obtained by collecting the remaining solid which was further purified by flash column chromatography. The silica was first neutralized with triethylamine and then eluted with CHCl_3_ mixed with methanol (1.0–4.0 %), Compound **L3** (80.0 mg, yield, 25.0 %) was obtained after recrystallization from DMF. ^1^H NMR (500 MHz, CDCl_3_) δ 8.46 (d, *J*=6.1 Hz, 8H), 7.48 (m, 32H), 7.36 (d, *J*=6.2 Hz, 8H), 7.27 (d, *J*=16.2 Hz, 4H), 7.13 (d, *J*=16.3 Hz, 4H), 7.06 (d, *J*=16.3 Hz, 4H), 6.99 (d, *J*=16.2 Hz, 4H), 2.17 (s, 12H). ^13^C NMR (126 MHz, CDCl_3_) δ 149.58, 149.16, 145.40, 138.13, 135.27, 133.32, 129.03, 127.71, 127.53, 126.96, 126.73, 125.52, 125.42, 121.14, 45.91, 39.40. *λ*_max_ (ϵ, cm^−1^ M^−1^)=364 nm (1.55×10^5^). ESI‐MS: *m*/*z* [M+H]^+^ calcd. for C_94_H_77_N_4_: 1262.64, found: 1263.00, correct isotope distribution.

**Formation of 10‐DIA, 16‐DIA, and 32‐DIA**: In a 5.0 mL vial, the ligand was dissolved in DMF (3.5 mg, 0.0048 mmol of **L1** in 1.0 mL DMF for the formation of **10‐DIA**; 3.0 mg, 0.0026 mmol of **L2** in 1.0 mL of DMF for the formation of **16‐DIA**; 2.5 mg, 0.0020 mmol of **L3** in 0.6 mL of DMF for the formation of **32‐DIA**). Subsequently, 3 μL, 2 μL, and 5 μL of HCl (37 %) was added to DMF solutions containing **L1**, **L2**, or **L3**, respectively. The metal salt solution was prepared by dissolving CuCl_2_ (13.4 mg, 0.099 mmol) in DMF (5.0 mL). This solution was added to the solutions containing **L1**(0.96 mL), **L2** (0.52 mL), or **L3** (0.4 mL). The vials were sealed and heated in an oven at 105 °C for 24 h (**10‐DIA**), 16 h (**16‐DIA**) and 48 h (**32‐DIA**). The reaction mixtures were allowed to attain room temperature by decreasing the temperature (10 °C/hour) of the oven by a controller (Lae Electronic, two‐channel universal controller, AC1‐5). The precipitates were separated from the solutions by centrifugation, and then washed with methanol, affording **10‐DIA** (green), **16‐DIA** (light yellow), and **32‐DIA** (dark red) in a yield of 50–70 %. The latter sample contains both **32‐DIA** and **L3** (needles).

Deposition Number(s) 2081776 (**10‐DIA**) and CCDC 2081777 (**16‐DIA**) and 2081778 (**32‐DIA**) contain(s) the supplementary crystallographic data for this paper. These data are provided free of charge by the joint Cambridge Crystallographic Data Centre and Fachinformationszentrum Karlsruhe Access Structures service.

## Conflict of interest

The authors declare no conflict of interest.

1

## Supporting information

As a service to our authors and readers, this journal provides supporting information supplied by the authors. Such materials are peer reviewed and may be re‐organized for online delivery, but are not copy‐edited or typeset. Technical support issues arising from supporting information (other than missing files) should be addressed to the authors.

Supporting Information

## Data Availability

The data that support the findings of this study are openly available in ChemRxiv at https://doi.org/10.26434/chemrxiv‐2021‐bf301, reference number 2021.
