# Supplementary material for: Chiral Motifs in Highly Interpenetrated Metal–Organic Frameworks Formed from Achiral Tetrahedral Ligands
Source: Chemistry. 2022 Aug 3;28(54):e202201108. doi: 10.1002/chem.202201108 (PMC9804673; doi:10.1002/chem.202201108)
Supplement: Supplementary file 1 — Supporting Information [file CHEM-28-0-s001.pdf]

Chemistry–A European Journal

Supporting Information

Chiral Motifs in Highly Interpenetrated Metal–Organic Frameworks Formed from Achiral Tetrahedral Ligands

Qiang Wen, Maria Chiara di Gregorio, Linda J. W. Shimon, Iddo Pinkas, Naveen Malik, Anna Kossoy, Eugeny V. Alexandrov, Davide M. Proserpio,* Michal Lahav, and Milko E. van der Boom*

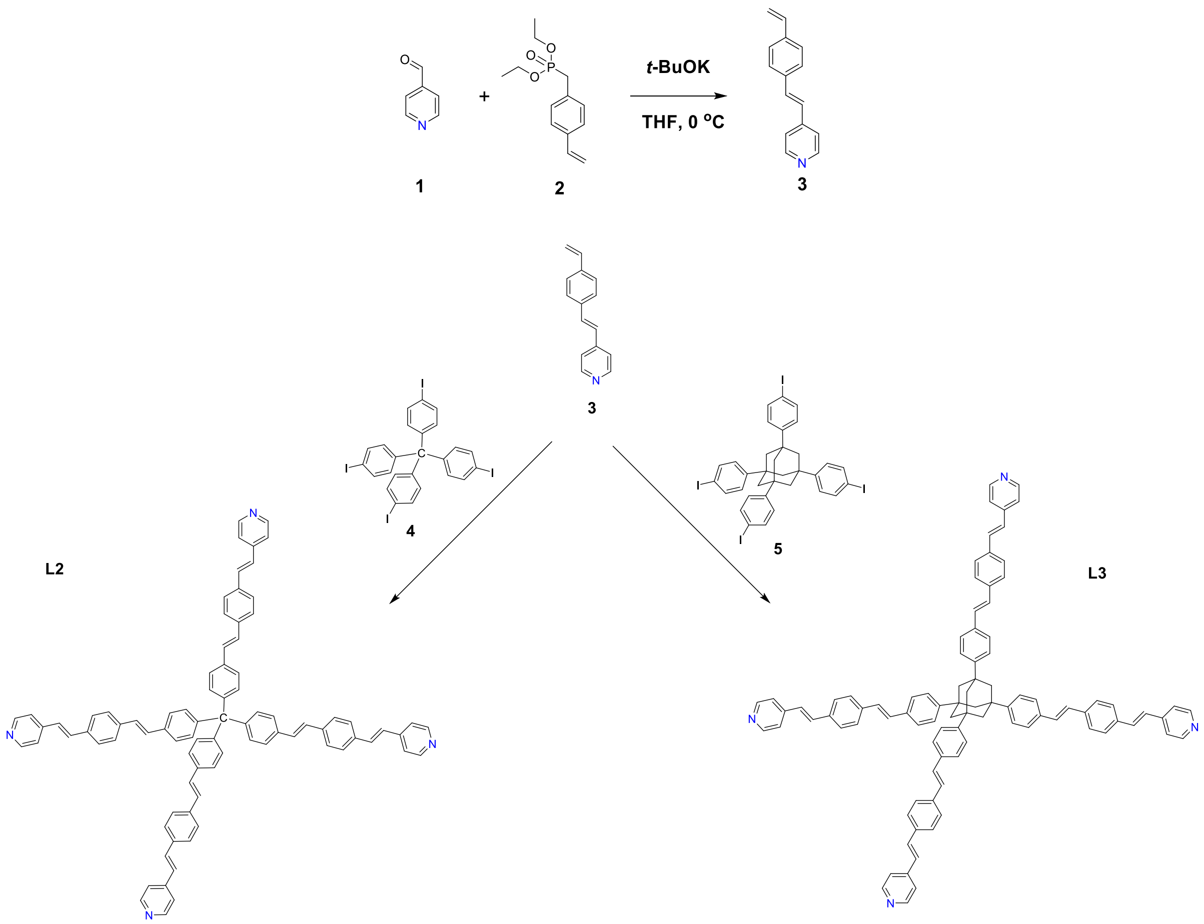

Scheme S1. Synthetic procedures for ligands **L2** and **L3**. Compounds **2**[S1], **4**[S2] and compound **5**[S3] were prepared according to literature procedures.

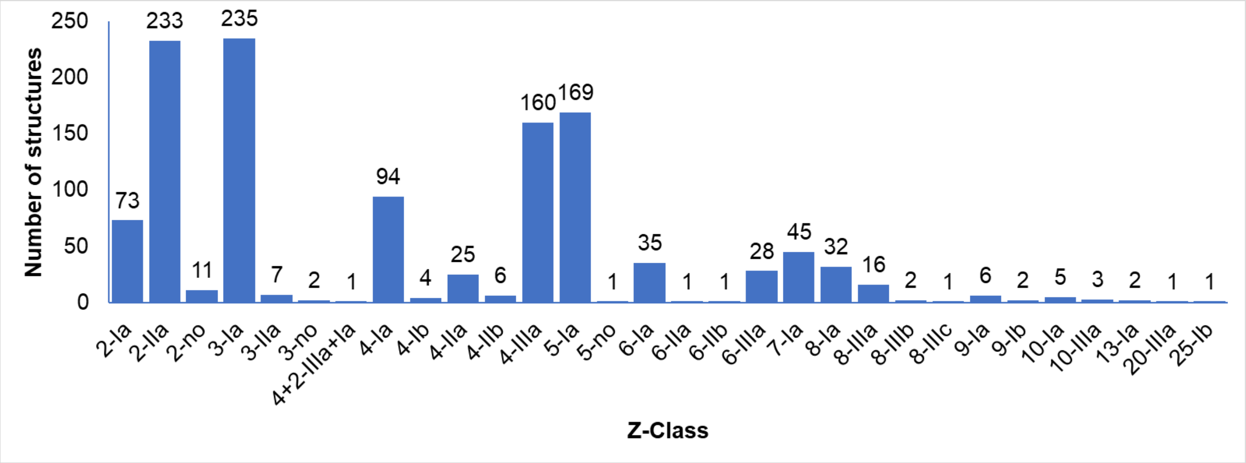

Figure S1. The distribution of 1202 coordination polymers (CSD release Nov. 2021, update Feb 2022) of topology **dia** in the standard representation over degrees and classes of interpenetration (the x-no do not belong to any class because they are rare cases of non-equivalent interpenetration)

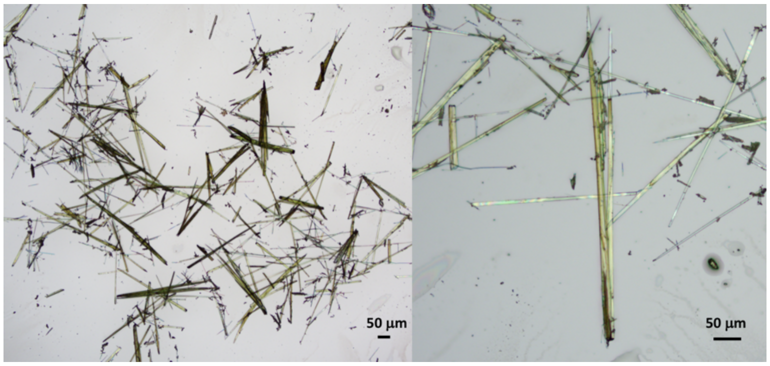

Figure S2. Optical microscopy images of **10-DIA**.

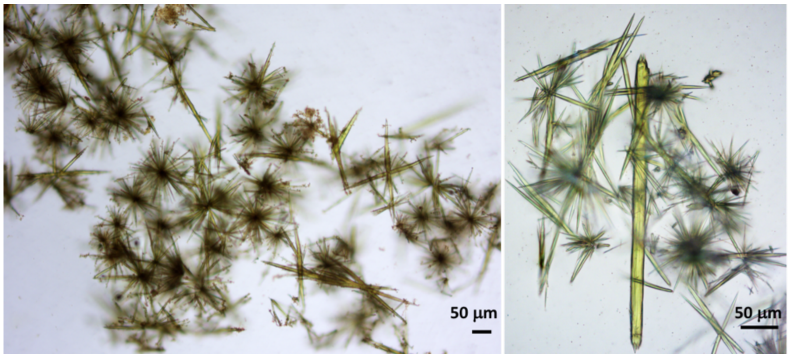

Figure S3. Optical microscopy images of **16-DIA**.

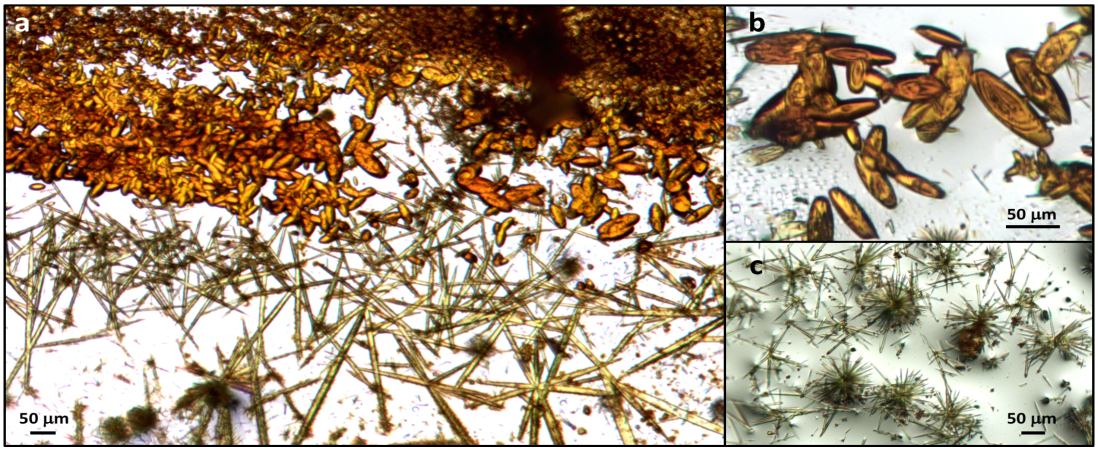

Figure S4. Optical microscopy images of **32-DIA**: red crystals in (a) and (b). The greenish materials shown in (a) and (c) are poorly diffracting and analyzed by microRaman spectroscopy.

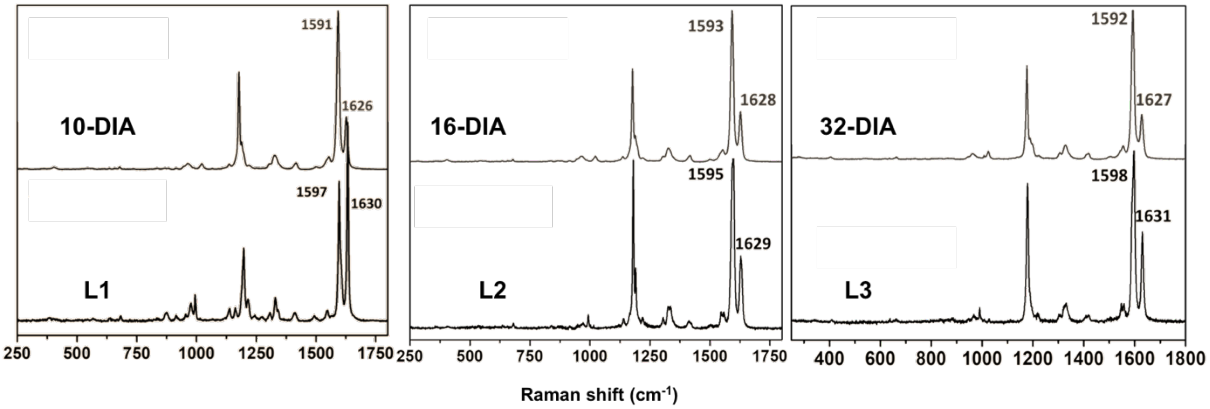

Figure S5. Representative microRaman spectra of DIA MOFs (top) and their corresponding ligands (bottom). The linewidths of the ligands are slightly narrower as the molecules are unconstrained while in the crystal state the bonds are restricted and the long range order is lower.

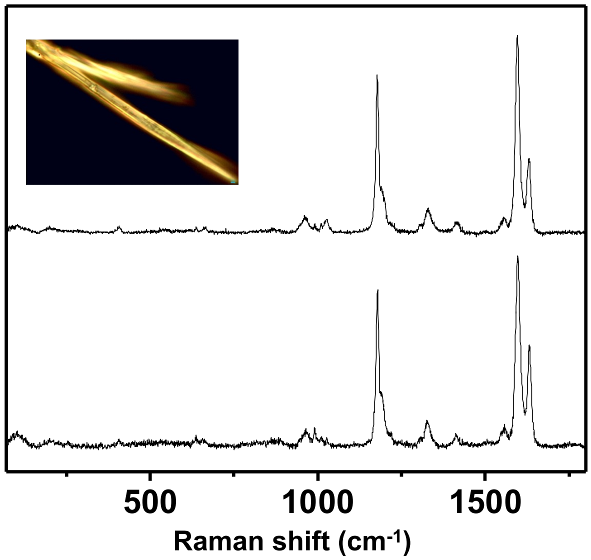

Figure S6. Representative microRaman spectra of the rod-like structures observed along with **32-DIA** (top) and of the powder of **L3** (bottom). Inset: optical microscopy image of the analyzed structure.

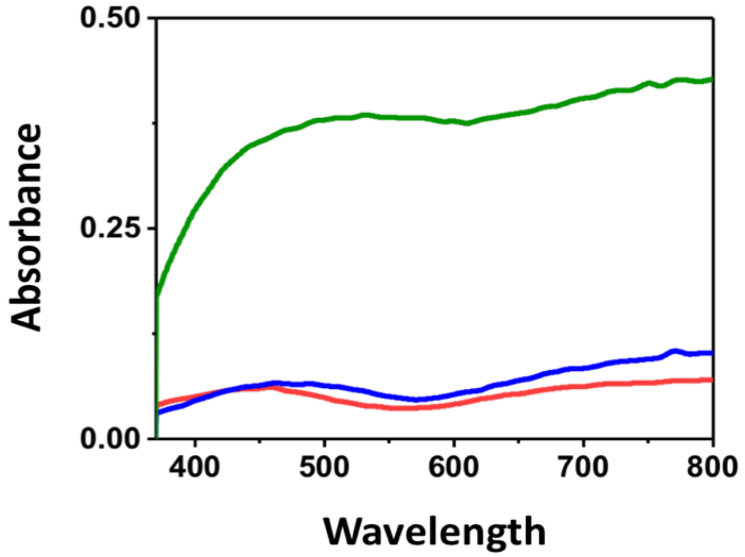

Figure S7. UV/Vis absorption spectra of **10-DIA** (red), **16-DIA** (blue) and **32-DIA** (green).

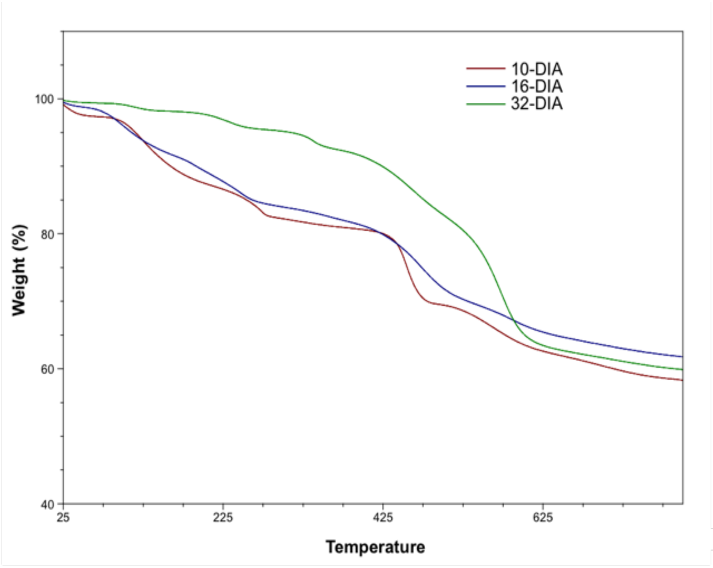

Figure S8. Thermogravimetric (TGA) curves under N_2.

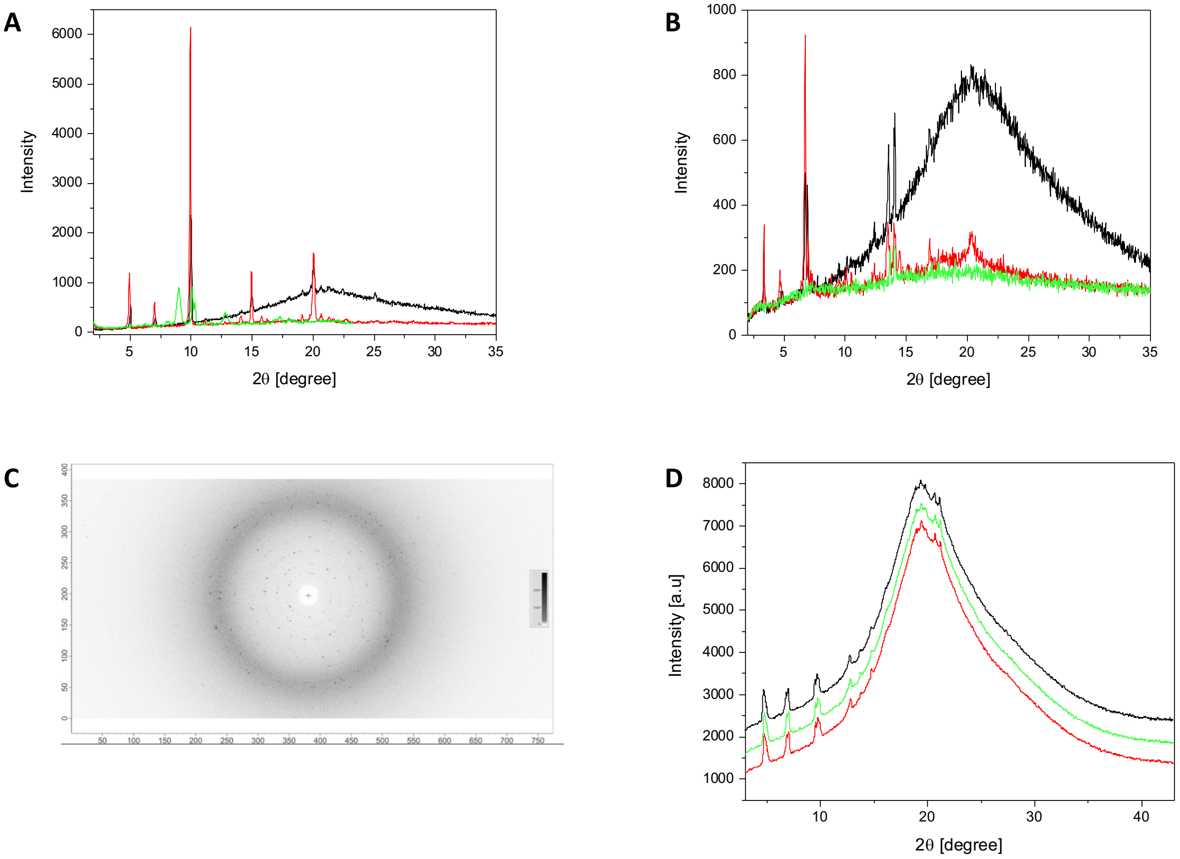

Figure S9. Representative Powder X-ray diffraction (PXRD) data. (A) **10-DIA** and (B) **16-DIA** in the presence of DMF. A fresh sample is exposed to X-ray radiation for 38 min. during each scan and the three consecutive scans are shown in the order black, red and green In the first (black) scan the large amount of solvent produce the large unresolved peak, (C,D) WAXS measurements of **10-DIA**. The samples were contained in a quartz capillary with DMF. (C) diffraction pattern, (D) Stability measurements: The same spot was measured 3 times: red: 2 h, green: 4 h, blue: 6 h (in total 6 hours of exposure to X-ray radiation). No change is evident. The intense bands around 2θ = 20 degrees are from the quartz capillary, also scattering from the mother solution contributes to the background.

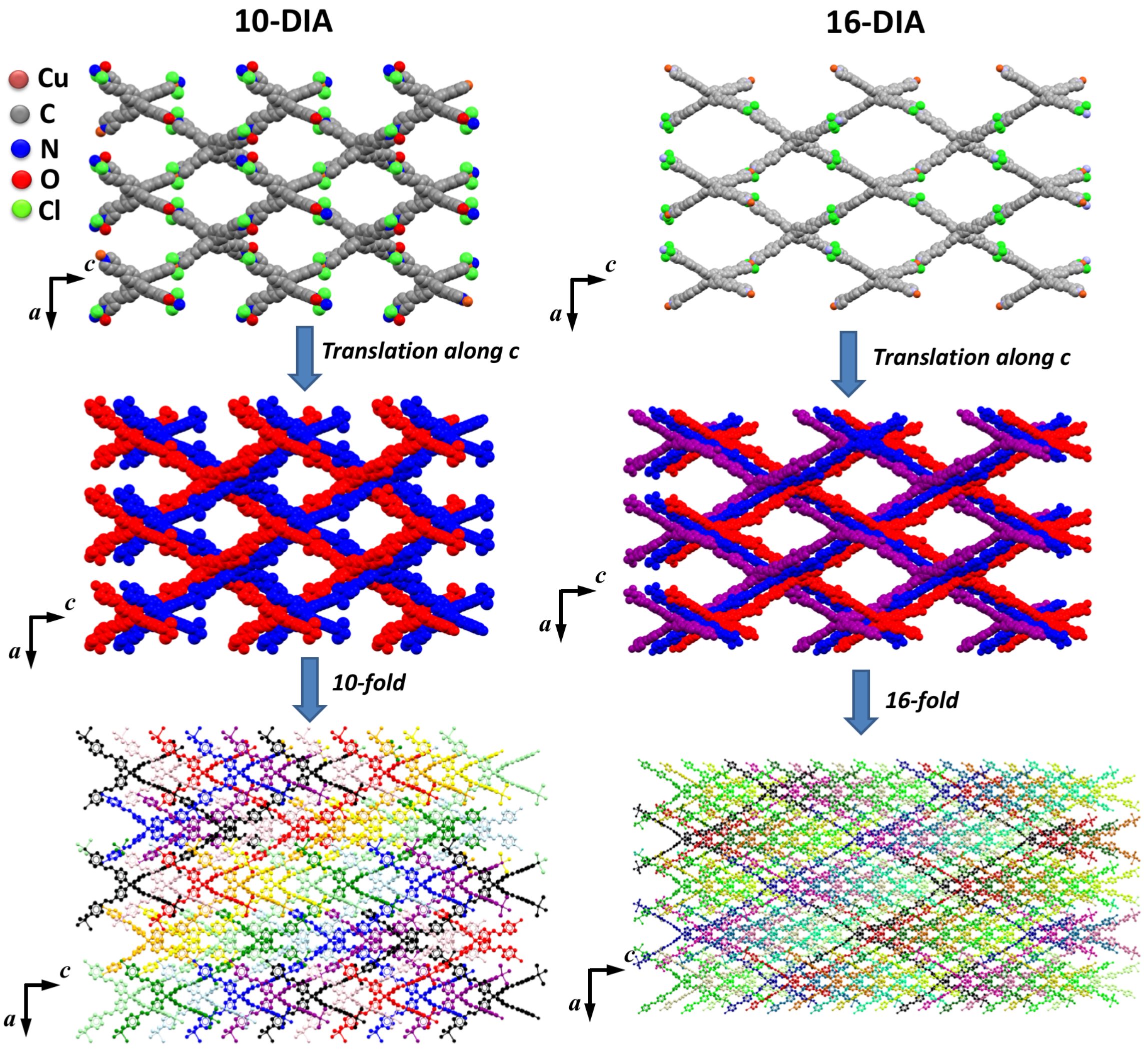

Figure S10. Interpenetration of **10-DIA** (left) and **16-DIA** (right): translation along *c* axis. Each diamondoid network is indicated with a color.

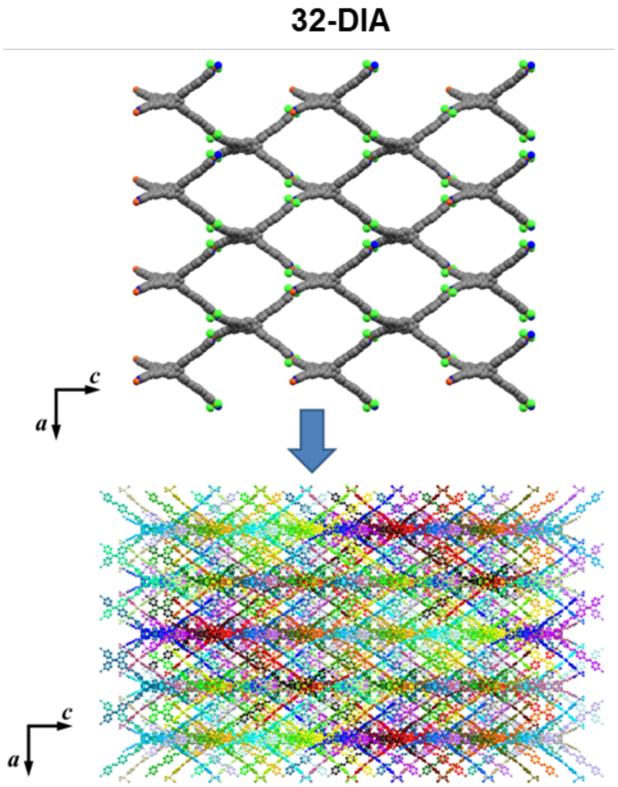

Figure S11. Interpenetration of **32-DIA**: a combination of a 4-fold screw axis and glide planes. Each diamondoid network is indicated with a color.

Figure S12. ToposPro outputs for **10-DIA**, **16-DIA** and **32-DIA**.

#########
10-DIA
#########

3D framework with ZA
There are 10 interpenetrating nets
FIV: Full interpenetration vectors

[0,0,1] (7.64A)

PIC: [0,0,10][1,1,0][1,0,5] (PICVR=10)

Zt=10; Zn=1
Class Ia Z=10

Point symbol for net: {6^6}
4-c net; uninodal net
Topological type: dia;

#########
16-DIA
#########

3D framework with ZA
There are 16 interpenetrating nets
TIV: Translating interpenetration vectors

[0,0,1] (15.11A)

NISE: Non-translating interpenetration symmetry elements
--
1: n[1,0,0]
2: n[0,1,0]
--
PIC: [0,0,8][1,1,0][1,0,4] (PICVR=8)

Zt=8; Zn=2

Class IIIa Z=16[8*2]
PIC could not be chosen for all sets of nets

Point symbol for net: {6^6}
4-c net; uninodal net
Topological type: dia

#########
32-DIA
#########

There are 32 interpenetrating nets
TIV: Translating interpenetration vectors

[1/2,1/2,1/2] (29.46A)
[1/2,1/2,-1/2] (29.46A)
[1/2,-1/2,1/2] (29.46A)
[1/2,-1/2,-1/2] (29.46A)

PISE: Partial interpenetration symmetry elements
--
1: -1; nets: 4
2: 2[1,-1,0]; nets: 3
3: 2[1,0,0]; nets: 4
4: 2[0,1,0]; nets: 4
5: d[1,-1,0]; nets: 2
--
PIC: [4,4,4][1,1,0][1,0,2] (PICVR=8)

Zt=8; Zn=4(2*2)

Class IIIc Z=32[8*(2*2)]
PIC could not be chosen for all sets of nets

Point symbol for net: {6^6}
4-c net; uninodal net

Topological type: dia

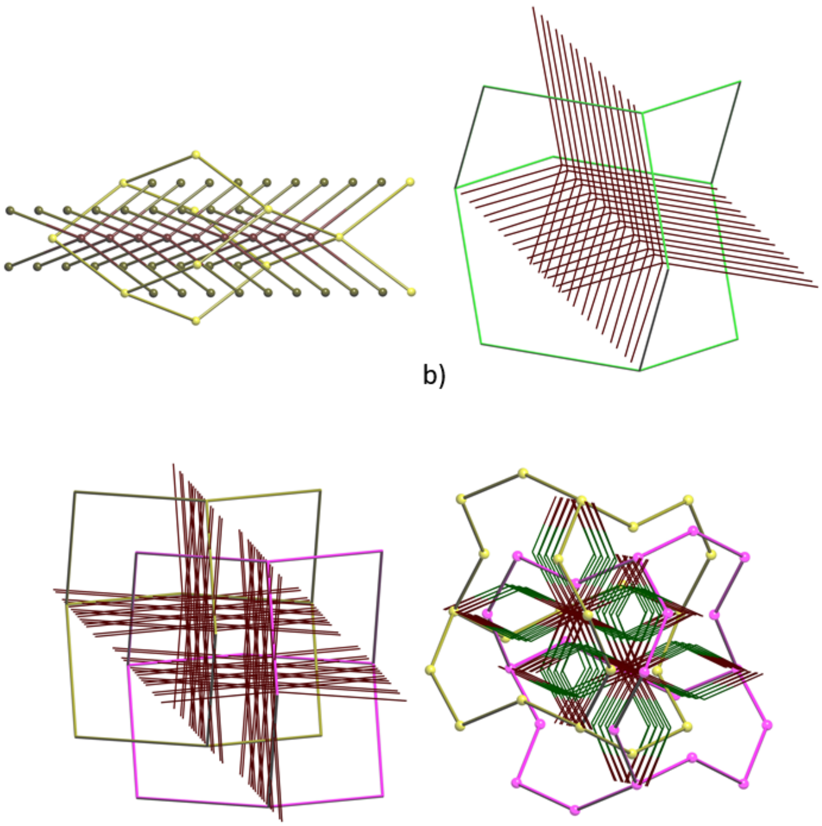
a) b)
c)

Figure S13. “Normal mode” of interpenetrating **dia** nets in **10-DIA** (a) **16-DIA** (b) and abnormal mode in **32-DIA** (c) with straight intersecting edges on the left, and with the observed not-intersectign bent edges (147°) on the right.

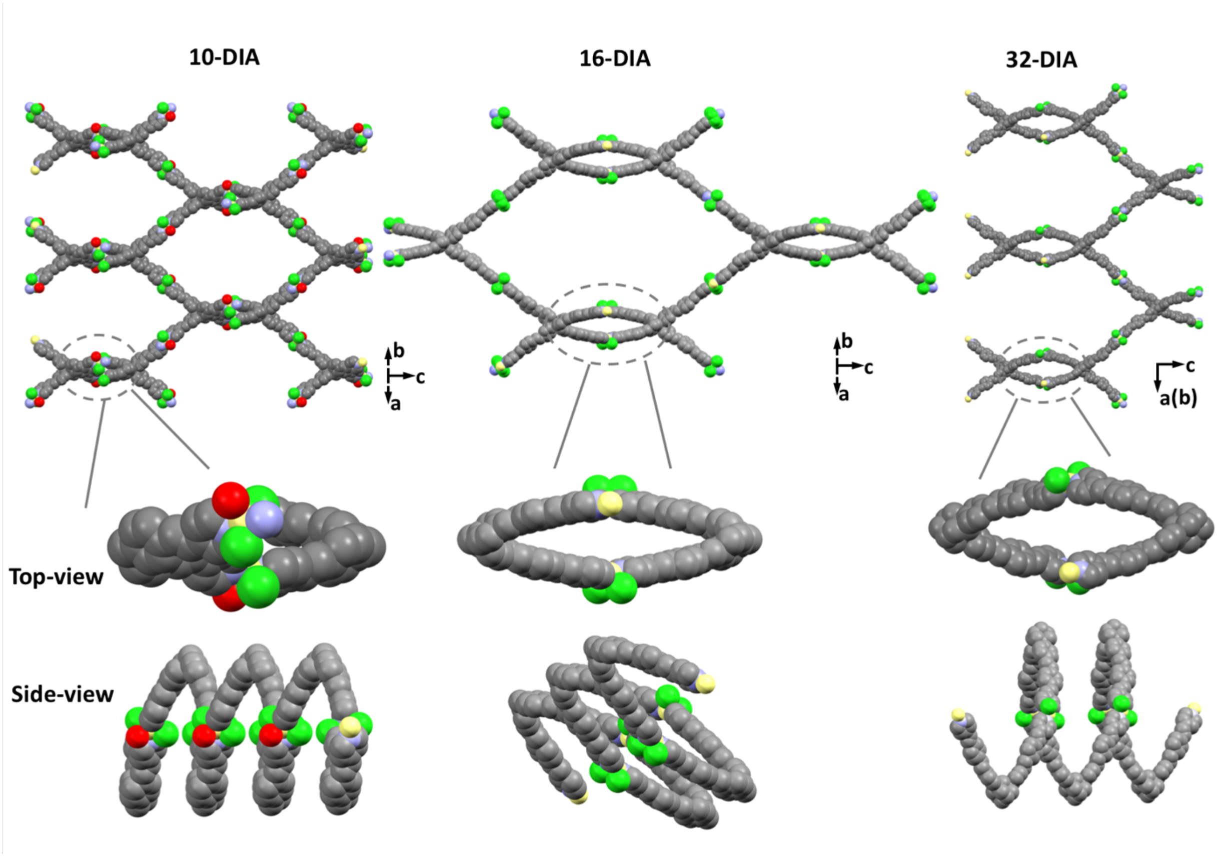

Figure S14. Helical structures with the left-handed handedness in a diamondoid network of the three MOFs. Color code is yellow for copper, red for oxygen, green for chlorine, violet for nitrogen and grey for carbon.

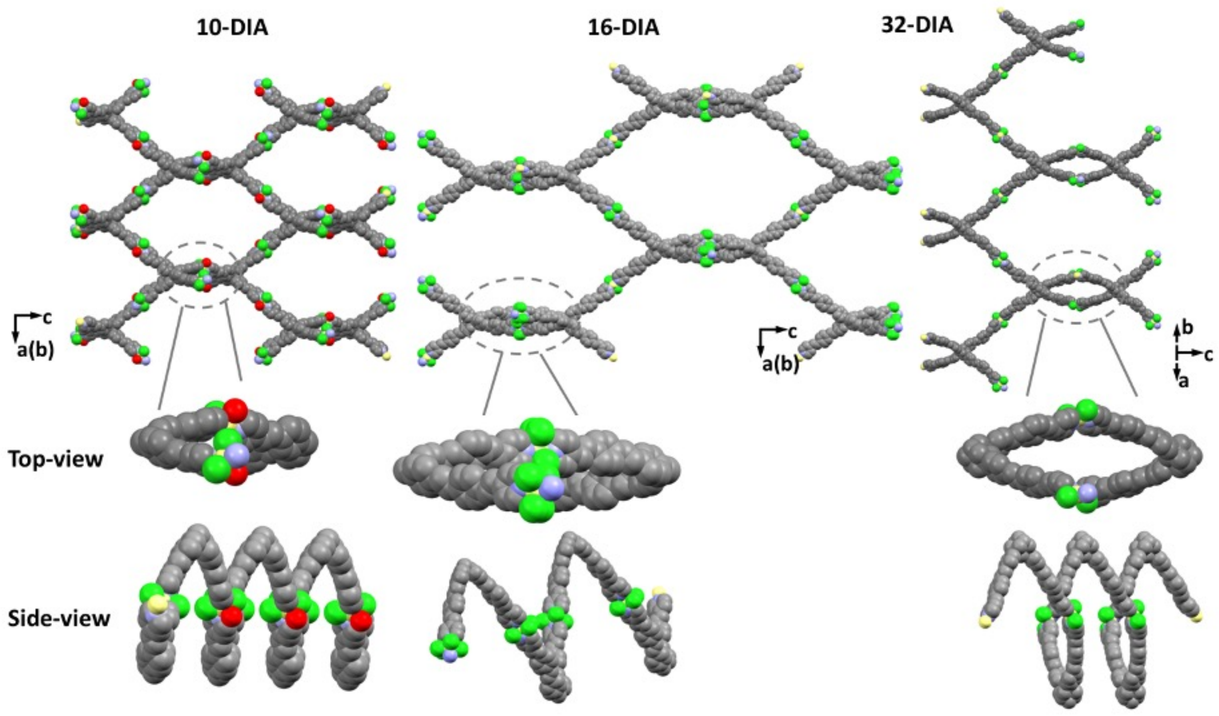

Figure S15. Helical structures with the right-handed handedness in a diamondoid network of the three MOFs. Color code is yellow for copper, red for oxygen, green for chlorine, violet for nitrogen and grey for carbon.

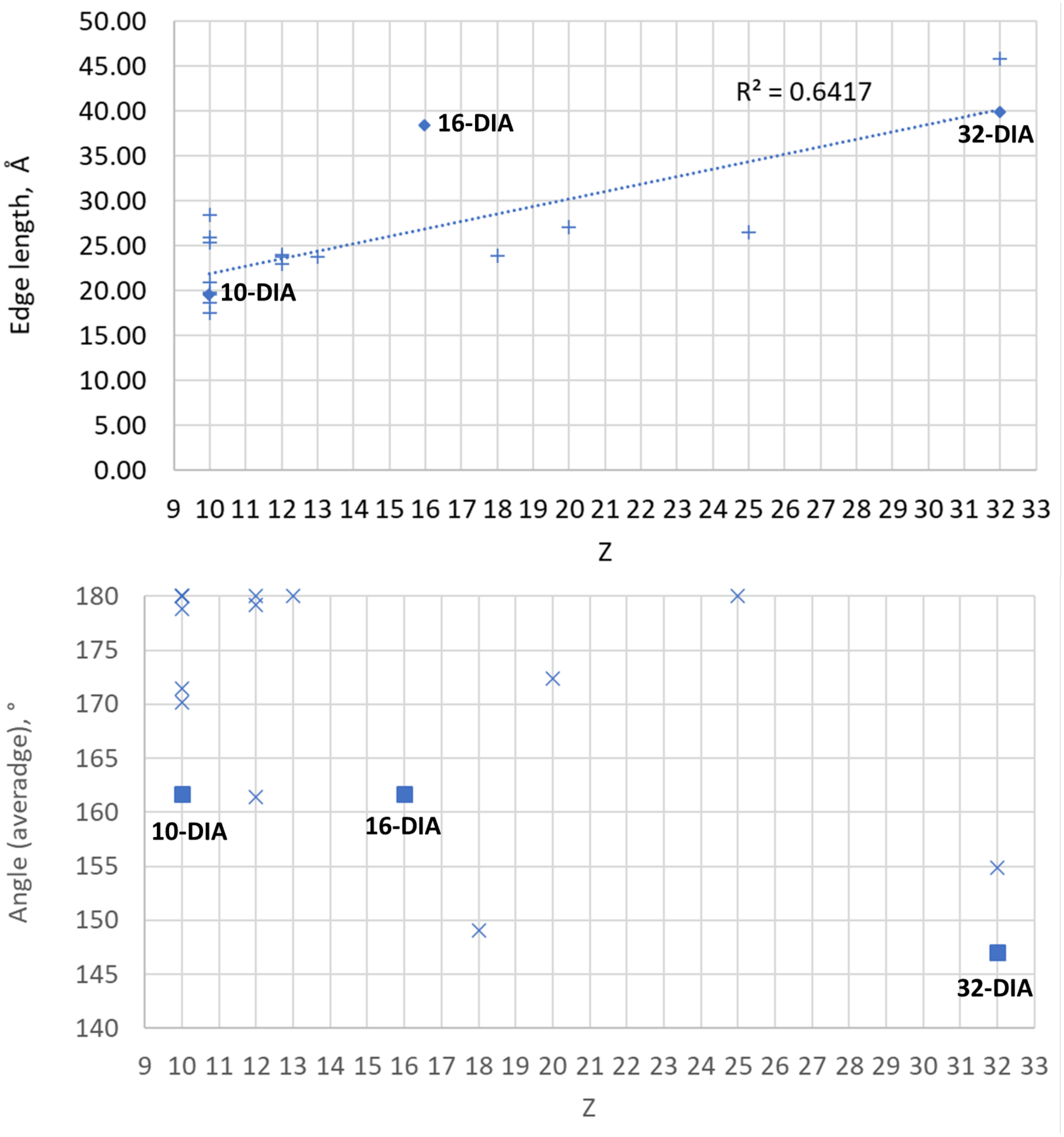

Figure S16. The relation of the average edges length (top) and angle of ligand bending (bottom) to degree of interpenetration for 17 known and 3 new highly interpenetrating structures.

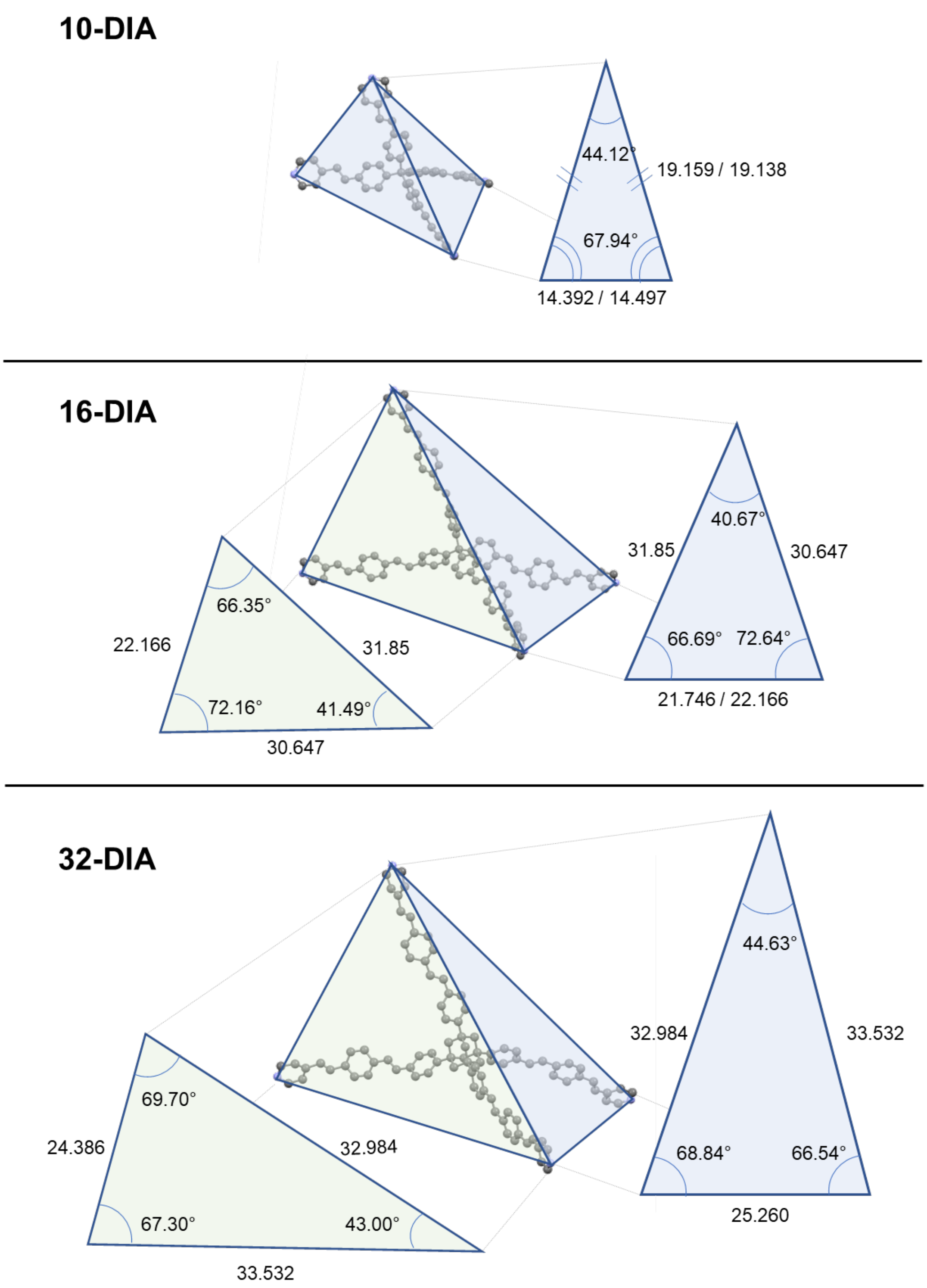

Figure S17. L1, **L2** and **L3** in the crystal structures of **10-DIA** (top), **16-DIA** (center) and **32-DIA** (bottom), respectively.

Table S1. Characteristics of reported structures with highly interpenetrated underlying **dia** net (white) and MOFs described in this work (yellow).

Refcode	Ref.	Space Group	Max symm SPGR & Transitivity [pq]	Accessible Channels	Distinct mode of interpe-netration	Representation	Average Edges length Å	Average Angle at middle of edge, °	Degree of Interpenetration Z	Class
FUXCOS	S4	*Fddd*	$P4_2/nnm$ [11] [a]	filled by anions	**dia-c10**	standard	19.55	171.5	10[5*2]	IIIa
PUQDOW	S4	*Pbca*	$P4_2/nnm$ [11] [a]	filled by anions	**dia-c10**	standard	19.4(1)	170.1(2)	10[5*2]	IIIa
XISXAY	S5	*Pnnn*	$P4_2/nnm$ [11] [a]	filled by anions	**dia-c10**	standard	19.80	180	10	Ia
FOKZEM	S6	*C2/c*	$I4_1/acd$ [11] [b]	no	**dia-c10****	standard	15.2, 19.7	180	10[5*2]	IIIa
VOTQAY	S7	$P4_2/n$	$P4_2/n$ [11] [b]	no	**dia-c10***	standard	20.90	180	10	Ia
WODYIZ	S8	*P1*	$P4_2/n$ [11] [b]	no	**dia-c10***	standard	16.7, 20.4	178.8(8)	10	Ia
SUFBAY	S9	$P4_2/n$	$P4_2/n$ [11] [b]	yes	**dia-c10***	standard	25.95	180	10	Ia
MOXPUL	S10	$P4_2/n$	$P4_2/nnm$ [11] [a]	yes	**dia-c10**	2-c Cluster $Cu_2(Ac)_4$ paddle-wheel	28.36	180	10	Ia
**10-DIA**		*P-4*	$P4_2/nnm$ [11] [a]	yes	**dia-c10**	standard	25.31	161.6	10	Ia
EJISAS	S11	*Pnna*	*Pban* [11] [c]	yes	**dia-c12***	Cluster 4-c SBU $Cu_2(SO_4)_2$	23.7(3)	180	12[6*2]	IIIa
KOBFEN	S12	$Pna2_1$	*Pban* [11] [c]	yes	**dia-c12***	Cluster 4-c SBU $Cu_2(SO_4)_2$	24.0(5)	179.2(1)	12[6*2]	IIIa
OMUNER09	S13	$P4_2/nbc$	*P4/nbm* [11] [a]	yes	**dia-c12**	H-bonds	22.9	161.4	12[6*2]	IIIa
UKEBOD	S14a	*C2/c*	*C2/c* [12] [c]	filled by anions	**dia-c13***	standard	23.80(1)	180	13	Ia
ZALVIV	S14b	$P2_1/c$	*C2/c* [12] [c]	filled by anions	**dia-c13***	standard	23.89(1)	180	13	Ia
**16-DIA**		*P-4n2*	*P4/nbm* [11] [a]	yes	**dia-c16**	standard	38.3(2)	161(5)	16[8*2]	IIIa
NUCJEB	S15	$I4_1/acd$	$I4_1/acd$ [11] [c] bent edges	no	**dia-c18***	H-bonds	23.82	149.04	18[(3*3)*2]	IIIb
KUVMEV	S16	$I4_1/a$	$I4_1/acd$ [11] [c]	no	**dia-c20***	standard	27.07	172.2(1)	20([5*2]+[5*2])	IIIa
ZEFHEZ	S17	$I4_1/a$	$I4_1/a$ [11] [c]	no	**dia-c25***	standard	26.47	180	25(5*5)	Ib
HOPQAH	S18	$I4_1/acd$	$I4_1/acd$ [11] [c]	yes	**dia-c32***	H-bonds	45.74	154.9	32[(4*4)*2]	IIIb
**32-DIA**		$I4_1/acd$	$I4_1/acd$ [11] [c] bent edges	yes	**dia-c32****	standard	39.84	147.0	32[8*(2*2)]	IIIc

[a] ref. S19 [b] ref. S20 [c] ref. S21

Table S2. Elemental analysis (EA). Weight percentage of the elements constituting bulk samples and their estimated formula. Weight percentage of the elements and formula derived from single-crystal X-ray data are reported for comparison.[a]

**10-DIA**	EA in wt%	C, 64.20; H, 3.82; N, 5.65; Cu, 8.86; Cl,3.72; O,13.75
	wt% derived from X-ray formula	C, 61.34; H, 4.27; N, 5.40; Cu, 12.25; Cl, 13.66; O, 3.08
	EA-derived formula	$C_{76.4}H_{54.1}N_{5.7}Cu_2Cl_{1.4}O_{12.3}$
	X-ray derived formula	$C_{53}H_{48}N_4Cu_2Cl_4O_2$
	Approximate content of solvent in wt%	14.7 for formula **10-DIA**·1.7DMF·8.6EtOH
**16-DIA**	EA in wt%	C, 72.31; H, 4.82; N, 5.20; Cu, 7.73; Cl, 9.30
	wt% derived from X-ray formula	C, 72.18; H, 4.85; N, 3.96; Cu, 8.99; Cl, 10.03
	EA-derived formula	$C_{100.3}H_{79.7}N_6Cu_2Cl_{4.3}O_{0.7}$
	X-ray-derived formula	$C_{85}H_{68}N_4Cu_2Cl_4$
	Approximate content of solvent in wt%	9.4 for formula **16-DIA**·2DMF

[a] The electron count observed by either Platon SQUEEZE or Olex2 confirms the presence of solvent molecules in the crystal structures. For **10-DIA** an electron count of 766 is observed which is comparable to 21 molecules of DMF. The electron count given by Olex2 for **16-DIA** is 3860, suggesting the presence of 96 molecules of DMF in the unit cell. The electron count for **32-DIA** is 1480, is consistent with the presence of 40 molecules of DMF in the unit cell. Bulk samples for TGA-MS and EA analyses were dried under high vacuum, resulting in lower amounts of solvents. Crystals for single crystal X-ray analysis were not washed with ethanol.

Table S3. Crystal data and structural refinement of **10-DIA**, **16-DIA**, and **32-DIA**.[a]

	**10-DIA**	**16-DIA**	**32-DIA**
CIF file	V384h	V372-cl	V381n-sr
CCDC	2081776	2081777	2081778
Crystal description	needle	needle	prism
Crystal size mm	0.250 × 0.020 × 0.020	0.250 × 0.020 × 0.020	0.080 × 0.060 × 0.060
Diffractometer	Rigaku XtaLabPro	Rigaku XtaLabPro	Rigaku XtaLabPro
Empirical formula	$C_{53}H_{44}Cl_4Cu_2N_4O_2$	$C_{85}H_{68}N_4Cu_2Cl_4$	$C_{47}H_{38}Cl_2CuN_2$
Formula weight (g/mol)	1037.80	1414.33	765.23
Temperature (K)	100	100	100
Wavelength (Å)	1.54184	1.54184	0.80
Space group	P-4	P-4n2	I 4_1/acd
a (Å)	23.502(3)	33.2590(12)	35.448(5)
b (Å)	23.502(3)	33.2590(12)	35.448(5)
c (Å)	7.6366(8)	15.1125(5)	30.968(6)
α, β, γ°	90,90,90	90,90,90	90,90,90
Volume ($Å^3$)	4218.1(13)	16716.9(13)	38913(13)
Z	2	4	32
Density calculated (Mg/m^3)	0.817	0.562	1.045
Absorption coefficient (mm^{-1})	1.991	1.059	0.897
F(000)	1064	2928	12704
Theta range for data collection (°)	2.659 to 42.605	3.2120 to 46.8910	3.5010 to 45.5810
Reflection collected (Unique)	73126 (9430)	27345 (9316)	158700 (10011)
R_{int}	0.0773	0.0910	0.0499
Completeness %	99.0	98.8	99.7
Index range *h*,*k*,*l*	-20, 20; -20, 16, -6, 6	-31,31; -18, 31; -12, 14	-28, 32;-32, 33;-29, 29
Data/restraints/parameters	2935 / 273 / 258	7515 / 203 / 423	4429 / 32 / 470
Goodness-of-fit on F^2	1.181	0.981	1.995
Final *R* [I>2σ(I)]	R1 = 0.0926<br>wR2 = 0.2028	R1 = 0.0874<br>wR2 = 0.2381	R1 =0.1604<br>wR2 = 0.4396
R (all data)	R_1 = 0.2576<br>wR_2 = 0.2702	R_1 = 0.1050<br>wR_2 = 0.2551	R_1 = 0.1753<br>wR_2 = 0.4555
Flack parameter	0.13(5)	0.53(5)	-

Table S4. Solvent accessible volume and volume within contact surface areas of **10-DIA**, **16-DIA** and **32-DIA** and other reported crystals. All the parameters were calculated by Mercury 2020.1, employing a spherical probe of 1.2 Å radius and a grid spacing of 0.7 Å.

	**Solvent accesible volume ($Å^3$)**	**Solvent accesible volume (% unit cell)**	**Volume within contact surface ($Å^3$)**	**Volume within contact surface (% unit cell)**
**10-DIA** **10-fold dia MOF**	1332.2	31.6	2132.2	50.6
**16-DIA** **10-fold dia MOF**	7668.5	45.9	10554.9	63.1
**32-DIA** **32-fold dia MOF**	5315.4	13.7	11120.23	28.6
**OYEYOH** **54-fold srs MOF[S22]**	249.5	0.9	2040.0	7.1
**ZEFHEZ** **25-fold dia MOF[S17]**	116.2	2.6	523.7	11.6
**HOPQAH** **32-fold dia H-bonded supremolecular network[S18]**	3944.2	12.5	9507.2	30.1

References

[S1] C. Carbonneau, R. Frantz, J. O. Durand, G. F. Lanneau, R. J. P. Corriu, *Tetrahedron Lett.* **1999**, *40*, 5855–5858.

[S2] I. Aujard, J. P. Baltaze, J. B. Baudin, E. Cogné, F. Ferrage, L. Jullien, E. Perez, V. Prévost, Lin Mao Qian; O. Ruel, *J. Am. Chem. Soc.* **2001**, 123, 8177–8188.

[S3] V. R Reichert, L. J. Mathias, *Macromolecules* **1994**, *27*, 7015–7023.

[S4] N. R. Andreychuk, S. R. Allard, S. L. M. Parent, A. Assoud, C. D. MacKinnon, *Cryst.Growth Des.* **2015**, *15*, 4377.

[S5] L. Carlucci, G. Ciani, D. M. Proserpio, S. Rizzato, *Chem.-Eur.J.* **2002**, *8*, 1519.

[S6] L. L. Dang, J. Q. Li, S. J. Liu, M. B. Luo, F. Luo, *Inorg.Chem.Commun.* **2014**, *45*, 30.

[S7] X. M. Guo, L. Zhao, H. Y. Zou, Y. N. Yan, Y. J. Qi, Q. Wang, *Inorg.Chem.Commun.* **2015**, *54*, 57.

[S8] M. Ahmad, R. Katoch, A. Garg, P. K. Bharadwaj, *CrystEngComm* **2014**, *16*, 4766.

[S9] T. W. Tseng, T. T. Luo, C. C. Tsai, K. L. Lu, *CrystEngComm* **2015**, *17*, 2935.

[S10] P. E. Ryan, C. Lescop, D. Laliberte, T. Hamilton, T. Maris, J. D. Wuest, *Inorg.Chem.* **2009**, *48*, 2793.

[S11] Y. Hao, B. Wu, S. Li, C. Jia, X. Juang, X.-J. Yang, *CrystEngComm* **2011**, *13*, 215.

[S12] Y. F. Hsu, C. H. Lin, J.-D. Chen, J. C. Wang, *Cryst.Growth Des.* **2008**, *8*, 1094.

[S13] Y. G. Huang, Y.Shiota, M.-Y. Wu, S.-Q. Su, Z.-S. Yao, S. Kang, S. Kanegawa, G.-L. Li, S. Q. Wu, T.Kamachi, K.Yoshizawa, K.Ariga, M.-C. Hong, O.Sato, *Nat.Commun.* **2016**, *7*, 11564.

[S14] (a) J. J. Liu, Y. F. Guan, M. J. Lin, C. C. Huang, W. X. Dai, *Cryst.Growth Des.* **2016**, *16*, 2836. (b) X. Li, Y. Li, X. Yang, C. Yan, K. Zhang, X. Liang, J. Zhang, Y Gai, K. Xiong *Chem. Commun.*, **2021**, *57*, 12496-12499.

[S15] Y. B. Men, J. Sun, Z.-T. Huang, Q. Y. Zheng, *CrystEngComm* **2009**, *11*, 978.

[S16] F. L. Geyer, F. Rominger, M. Vogtland, U. H. F. Bunz, *Cryst.Growth Des.* **2015**, *15*, 3539.

[S17] Y. P. He, Y. X. Tan, J. Zhang, *CrystEngComm* **2012**, *14*, 6359.

[S18] S. A. Boer, P. X. Wang, M. J. MacLachlan, N. G. White, *Cryst.Growth Des.* **2019**, *19*, 4829.

[S19] C. Bonneau, M. O'Keeffe *Acta Cryst. A* **2015**, *71*, 82-91.

[S20] I. A. Baburin, *Acta Cryst. A* **2016**, *72*, 366-375.

[S21] I. A. Baburin, personal communication

[S22] H. Wu, J. Yang, Z. M. Su, S. R. Batten, J. F. Ma, *J. Am. Chem. Soc.* **2011**, *133*, 11406–11409.
